# The Screen for Cognitive Impairment in Psychiatry (German version, SCIP-G): Validation, dimensionality analysis and practical application in inpatient psychiatric treatment

**DOI:** 10.1192/j.eurpsy.2023.813

**Published:** 2023-07-19

**Authors:** A. Erfurth, G. Sachs

**Affiliations:** 1 1st Department of Psychiatry and Psychotherapeutic Medicine, Klinik Hietzing; 2 Medical University of Vienna, Vienna, Austria

## Abstract

**Introduction:**

Psychiatric disorders are often characterised by cognitive impairment. The Screen for Cognitive Impairment in Psychiatry (SCIP) was developed for routine screening of psychiatric patients and is available in several languages.

**Objectives:**

Using the German version (SCIP-G), 3 studies were conducted: 1. feasibility, reliability, and validity of the SCIP-G were investigated [Sachs et al. Schizophr. Res. Cogn. 2021: 25, 100197], 2. a confirmatory factor analysis was performed [Sachs et al. Schizophr. Res. Cogn. 2022: 29, 100259], and 3. patients with psychotic, bipolar affective, and depressive disorders were assessed before and after standard inpatient treatment including cognitive remediation.

**Methods:**

Study 1 included patients with schizophrenia or schizoaffective psychosis and thirty healthy controls matched for sex, age, and education. Data were collected at the Medical University of Vienna, Department of Psychiatry and Psychotherapy. In studies 2 and 3, patients from the Klinik Hietzing, 1st Department of Psychiatry and Psychotherapeutic Medicine, Vienna, Austria, were studied. In study 3, all patients received modern pharmacotherapy plus cognitive remediation using the COGPACK® software package version 6.06; based on the ICD-10 criteria for research, 54 patients received an F2 diagnosis (schizophrenia, schizotypal, and delusional disorders), 39 patients met criteria for bipolar disorder (F30 and F31), and 50 for depression (F32 and F33).

**Results:**

In Study 1, significant differences in cognitive performance were found between patients and healthy controls on both versions of the SCIP. The SCIP effectively discriminated between patients and the control group. In Study 2, a two-factor solution in which the Verbal Learning Test-Immediate Recall subtests, Delayed Recall Test of the VLT, and Working Memory Test loaded on the first factor and the Verbal Fluency Test and Psychomotor Speed Test subtests loaded on the second factor yielded good model fit (χ² = 6.7, df = 3, p = .08, χ²/df = 2.2). In Study 3, SCIP total score showed significant improvement after treatment in all three diagnostic groups (p < .001), with no statistically significant interaction between SCIP total score and diagnostic groups (p = .860).

**Conclusions:**

Our data indicate that the SCIP-G is a valid and reliable instrument for assessing cognitive impairment. Good model fit can be achieved with a two-factor solution for the SCIP. Our study is the first to perform a confirmatory factor analysis with the German SCIP version and to test its dimensional structure with a hypothesis-testing approach. Inpatient treatment consisting of pharmacotherapy and cognitive remediation improved cognitive deficits. This improvement in cognitive performance was observed to a similar extent in patients with psychotic disorders, bipolar disorder, and depression.

**Disclosure of Interest:**

None Declared

